# Estimation of total phenol and *in vitro* antioxidant activity of *Albizia procera* leaves

**DOI:** 10.1186/1756-0500-6-121

**Published:** 2013-03-27

**Authors:** Mahfuza khatoon, Ekramul Islam, Rafikul Islam, Aziz Abdur Rahman, AHM Khurshid Alam, Proma Khondkar, Mamunur Rashid, Shahnaj Parvin

**Affiliations:** 1 Department of Pharmacy, University of Rajshahi, Rajshahi-6205, Bangladesh; 2 Department of Pharmacy, International Islamic University of Chittagong, Chittagong, 4203, Bangladesh; 3 Department of Pharmaceutical and Biological Sciences, UCL School of Pharmacy, London, UK

**Keywords:** Antioxidants, Free radical scavenging, Phytochemical constituents, Total phenolic content

## Abstract

**Background:**

Research on natural products has gained a wide popularity due to the potential of discovering active compounds. The antioxidant properties contained in plants have been proposed as one of the mechanisms for the observed beneficial effect. Therefore, the present study investigated the antioxidant activity and total phenolic contents of various solvent extracts of *Albizia procera* leaves.

**Methods:**

Antioxidant activity of the methanol extract and its derived fractions petroleum ether (APP), carbon tetrachloride (APC), dichloromethane (APD), ethyl acetate (APE), and residual aqueous fraction (APA) of the leaves of *Albizia procera* was performed by *in vitro* chemical analyses. Total phenolic content of the APM and other five fractions were also determined. APM and its derived fractions were also subjected to preliminary phytochemical screening test for various constituents.

**Results:**

Phytochemical screening revealed the presence of saponins, steroids, tannins, glycosides and flavonoids in the extracts. Amongst the extracts, APE showed the highest total phenolic content (449.18 ± 18.41mg of gallic acid equivalent/g of extract). In DPPH (1,1-diphenyl-2-picrylhydrazyl) radical scavenging test, the IC_50_ value of APM, APP, APC, APD, APE and APA was 43.43, 63.60, 166.18, 41.15, 11.79, and 63.06 μg/mL, respectively. Therefore, among the APM and its derived fractions, APE showed the highest antioxidant activity which is comparable to that of standard ascorbic acid (AA) (IC_50_ 10.12 μg/mL). The total antioxidant capacity was found to be varied in different fractions. The reducing activity on ferrous ion was ranked as APE > APD > APM > APA > APC.

**Conclusion:**

The above evidences suggest that APE of *A. procera* leaf is a potential source of natural antioxidant and can be used to prevent diseases associated with free radicals.

## Background

Free radical is constantly generated in all living cells and is a part of normal cellular function. However, excess free radical originating from endogenous or exogenous sources are responsible for aging and causing various human diseases. Free radicals cause oxidative damage to different molecules, such as lipids, proteins and nucleic acids and thus are involved in the initiation phase of some degenerative diseases. Research has shown that free radical mediated oxidative stress is among the major causative factors in induction of many chronic and degenerative diseases including atherosclerosis, ischemic heart disease, ageing, diabetes mellitus, cancer, immunosuppression, neurodegenerative diseases and others [1]. Antioxidants prevent free radicals from doing harm to our DNA, proteins, and cells by donating electrons to stabilize and neutralize the harmful effects of the free radicals. This action helps in protecting the body from degenerative diseases. With that, the role of antioxidants has drawn much attention as a candidate to combat certain diseases and prevent the aging process [2]. An antioxidant can be defined as: any substance that when present in low concentrations compared to that of an oxidisable substrate significantly delays or inhibits the oxidation of molecules, by inhibiting the initiation or propagation of oxidizing chain reactions [3].

Ascorbic acid, carotenoids and phenolic compounds are naturally occurring effective antioxidants [4]. A great number of aromatic, medicinal, spice and other plants contain chemical compounds exhibiting antioxidant properties. Recently, there has been a great of interest in the therapeutic potential medicinal plants as antioxidants in reducing oxidative stress-induced tissue injury [5]. Various studies carried out on medicinal plants strongly supports the idea that plant constituents with antioxidant activity are capable of exerting protective effects against oxidative stress in biological systems [6]. In addition to above work, we studied several extracts of leaves of *Albizia procera* for the search of antioxidant activity from plant sources.

*A. procera* is a tree belongs to the family Fabaceae, widely distributed from India and Myanmar through Southeast Asia to Papua New Guinea and northern Australia. Leaves said to be insecticidal [7]. In folk medicine, bark is used for fish poison. Leaves are poulticed onto ulcers in India. Bark also considered useful in pregnancy and stomachache and is given with salt as a medicine for water buffalo [8]. The ethanolic extracts of bark showed signicicant anti-HIV-1 integrase activity [9]. The bark extract of *Albizia procera* showed potent DPPH scavenging activity [10]. However, still there is no report on the antioxidant activity of the leaves of this plant. Therefore, this study was conducted to investigate the antioxidant activities of extracts from the leaves of *Albizia procera,* particularly for finding new sources for natural antioxidants.

## Methods

### Chemicals and reagents

1,1-diphenyl-2-picrylhydrazyl radical (DPPH), Folin-Ciocalteu reagent were obtained from Sigma-Aldrich (St. Louis, USA). Methanol was bought from SIGMA® (Sigma- Aldrich®, St Louis, USA). Chloroform, dichloromentane, carbon tetrachloride, petroleum ether, gallic acid, quercetin, sodium carbonate (Na_2_CO_3_), ferric chloride (FeCl_3_), potassium ferricyanide [K_3_ Fe(CN)_6_], trichloroacetic acid (TCA), buffer and ascorbic acid were purchased from Merck (Darmstadt, Germany). All chemicals used were of analytical grade.

### Collection of plant material

Fresh leaves of the plants were collected in May, 2012 and identified by Botany Department, Rajshahi University, Bangladesh. A voucher specimen with accession no.3798 has been deposited in Bangladesh National Herbarium, Dhaka, Bangladesh.

### Preparation of plant extracts

The leaves were left to dry under shade, grinded and extracted with methanol by cold maceration for 7 days at room temperature. The extract was then filtered off through a cotton plug and finally through filter paper. The filtrate was concentrated using vacuum rotary evaporator at 50°C. The concentrated methanol extract was fractionated by the modified Kupchan partitioning method [11] into petroleum ether, dichloromethane, carbon tetrachloride, and ethyl acetate fractions. After that, all the extracts were stored in a refrigerator for further use.

### DPPH radical scavenging activity

The free radical scavenging activity of the extracts and ascorbic acid as positive control was measured in terms of hydrogen donating or radical-scavenging ability using the stable radical DPPH by the method described by Susanti *et al*. [12] with slight modifications. 2mL of each extract and control at various concentrations (100, 50, 25, 12.5, 6.25, 3.125, 1.625, and 0.812 μg/mL) were added to 3 ml of freshly prepared DPPH solution (0.004%) in methanol. The reaction was allowed for 30 min and absorbance was measured at 515 nm using a spectrophotometer (HACH 4000 DU UV – visible spectrophotometer). All experiments were repeated three times independently. The degree of decolorization of DPPH from purple to yellow indicated the scavenging efficiency of the extract. The percentage inhibition of DPPH free radical scavenging activity was calculated using the following equation:

$$\mathrm{Percent}\;\mathrm{inhibition}=\left[\left(A_{\mathrm{DPPH}}-A_{\mathrm{sample}}\right)/A_{\mathrm{DPPH}}\right]\times 100$$

Where:

A_DPPH_ = Absorbance of DPPH

A_sample_ = Absorbance of sample (extract/ascorbic acid)

The % inhibition data was then plotted against log concentration fitted in a graph and IC_50_ (half-maximal inhibitory concentration) value was calculated by linear regression analysis.

### Reducing power capacity

Reducing power of the extract was evaluated by Oyaizu method [13]. Different concentrations of leaves extract and ascorbic acid as standard (6.25, 12.5, 25, 50, and 100 μg/mL) in 0.25ml methanol were mixed with phosphate buffer (0.625 ml, 0.2 M, pH 6.6) and potassium ferricyanide [K_3_Fe (CN)_6_] (0.625 ml, 1% w/v). The mixture was vortex and incubated at 50°C for 20 min. After incubation, 0.625ml of 10% trichlorocacetic acid solution was added to each tube and the mixture was centrifuged at 3000 rpm for 10 minutes. 1.8 ml of the upper layer solution was mixed with equal volume of distilled water and 0.36 ml of ferric chloride solution (0.1% w/v) and the absorbance was measured at 700 nm. The reducing power of the extract was linearly proportional to the concentration of the sample. Phosphate buffer (P^H^ 6.6) was used as blank solution.

### Phosphomolybdate assay (total antioxidant capacity)

Total antioxidant activity of the fractions was evaluated by the phosphomolybdate method using AA as a standard [14]. The assay is based on the reduction of Mo (VI)-Mo (V) by the extract and subsequent formation of a green phosphate/Mo (V) complex at acidic pH. An aliquot of 0.3 mL extract was combined with 3 ml of reagent solution (0.6 M sulfuric acid, 28 mM sodium phosphate and 4 mM ammonium molybdate). The tubes containing the reaction solution were incubated at 95°C for 90 min. After the samples had cooled to room temperature, the absorbance of the solution was measured at 695 nm against blank using a spectrophotometer. Methanol (0.3 mL) in the place of extract is used as the blank. Ascorbic acid equivalents were calculated using standard graph of AA. The experiment was conducted in triplicates and values are expressed as equivalent of ascorbic acid in mg per g of extract.

### Estimation of total phenolic content

Total phenolic content of all the extracts was evaluated with Folin-Ciocalteu method [15]. Samples containing polyphenols are reduced by the Folin-Ciocalteu reagent there by producing blue colored complex. The phenolic concentration of extracts was evaluated from a gallic acid calibration curve. To prepare a calibration curve, 0.5mL aliquots of 12.5, 25, 50, 100, 200, and 400 μg/mL methanolic gallic acid solutions were mixed with 2.5 mL Folin–Ciocalteu reagent (diluted ten-fold) and 2.5 mL (75 g/L) sodium carbonate. After incubation at 25°C for 30 min, the quantative phenolic estimation was performed at 765 nm against reagent blank by UV Spectrophotometer 1650 Shimadzu, Japan. The calibration curve was constructed by putting the value of absorbance vs. concentration. A similar procedure was adopted for the extracts as above described in the preparation of calibration curve. All determinations were performed in triplicate. Total phenolic content was expressed as milligrams of gallic acid equivalent (GAE) per g of extract.

### Phytochemical screening of APM

Phytochemical screening was done as described by Dohou *et al*. [16]. Qualitative screening of APM and various fractions of *A. procera* leaves was performed for the identification of phytochemicals like saponins, tannins, glycosides, flavonoids, steroids and alkaloids.

### Statistical analysis

All analyses were carried out in triplicates. Data were presented as mean *±* SD. Free R-software version 2.15.1 (http://www.r-project.org/) and Microsoft Excel 2007 (Roselle, IL, USA) were used for the statistical and graphical evaluations.

## Results

### DPPH radical scavenging activity

**Figure 1:**
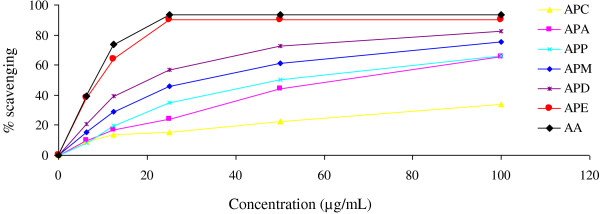
**Determination of DPPH radical scavenging activity of APM and its derived fractions (APP, APC, APD, APE and APA).** Each point represents the mean of three experiments. Data expressed as mean *±* standard deviation. APM = Methanol extract, APP = Petroleum ether fraction, APC = Carbon tetrachloride fraction, APD = Dichloromethane fraction, APE = Ethyl acetate fraction, and APA = Aqueous fraction.

DPPH radical scavenging based antioxidant potential of the extracts was evaluated using the parameter IC_50_. Here, IC_50_ means the concentration of antioxidant required for 50% scavenging of DPPH radicals in the specified time. *In vitro* antioxidant activities of all the extracts were measured with the standard antioxidant AA. The extracts had dose-dependent activity, *i.e.* DPPH scavenging activity increased proportionate to the increase in concentration of the extracts. Results are shown in Figure 1. The smaller IC_50_ values, the higher antioxidant activity of the plant extracts [17]. Table 1 shows the calculated IC_50_ values of the standard and extracts. Amongst the six extracts, APE revealed the highest scavenging activity (90.02%) at the concentration of 25 μg/mL with IC_50_ value of 11.79 μg/mL.

**Table 1:** **Free radical scavenging activity of standard (AA), APM and its derived fractions of *A. procera *leaves**

**Test samples**	**% Inhibition at 25 μg/mL**	**IC**_**50**_**values (μg/mL)**
AA	93.65	10.12
APM	51.30	43.43
APP	30.16	63.60
APD	36.09	41.15
APC	15.28	166.18
APE	90.02	11.79
APA	24.14	63.01

APM = Methanol extract, APP = Petroleum ether fraction, APC = Carbon tetrachloride fraction, APD = Dichloromethane fraction, APE = Ethyl acetate fraction and APA = Aqueous fraction.

### Ferric reducing capacity

**Figure 2:**
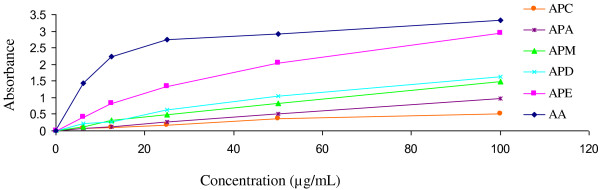
**Determination of ferrous reducing capacity of APM and its derived fractions (APC, APD, APE and APA).** Data expressed as mean *±* standard deviation. APM = Methanol extract, APC = Carbon tetrachloride fraction, APD = Dichloromethane fraction, APE = Ethyl acetate fraction, and APA = Aqueous fraction.

The reducing capacity of a compound indicates its potential antioxidant activity. Figure 2 shows the dose response curves for the reducing power of APM and its derived fractions (6.25–100 μg/ml). At the lowest concentration, the absorbance of APM, APC, APD, APE, APA and AA was 0.125, 0.0655, 0.2095, 0.4077, 0.0705 and 1.431, respectively. At the highest concentration, absorbance of the above extracts was 1.479, 0.499, 1.637, 2.949, 0.98 and 3.333, respectively. APP did not show any activity within the above range of concentrations and data are not shown here. The extracts displayed a concentration dependent increase in reducing power. The reducing power increased with increasing amount of the extracts. Higher absorbance of the reaction mixture indicates a higher reducing power. Thus, the present results showed that higher reducing power was evident in APE.

### Determination of total antioxidant activity

**Figure 3:**
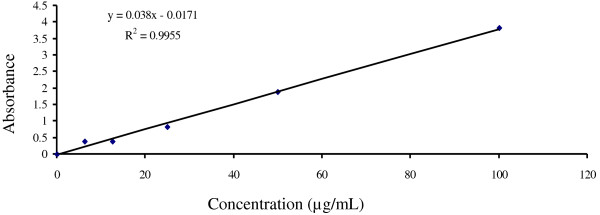
**Calibration curve of ascorbic acid.** Each point represents the mean of three experiments.

**Figure 4:**
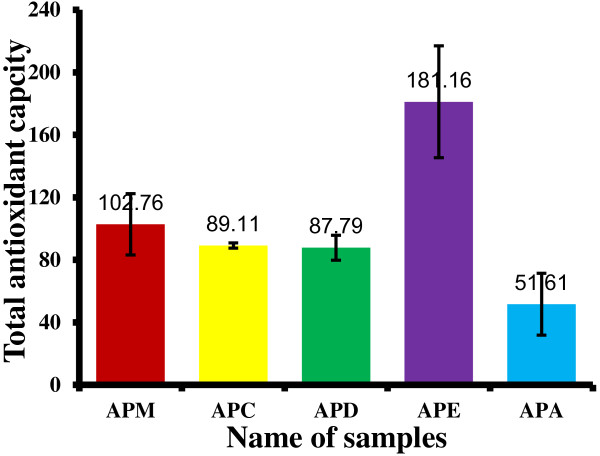
**Total antioxidant capacity of APM and its derived fractions (APC, APD, APE and APA).** Values expressed are mean ± standard deviation (n = 3). Total antioxidant capacity is expressed as mg of AA equivalent per gm of extract. APM = Methanol extract, APC = Carbon tetrachloride fraction, APD = Dichloromethane fraction, APE = Ethyl acetate fraction, and APA = Aqueous fraction.

Total antioxidant capacity of the extracts, expressed as the number of equivalent of AA was obtained from the calibration curve as shown in Figure 3. The phosphomolybdate method was based on the reduction of Mo (VI) to Mo (V) by the antioxidant compound and the formation of a green phosphate/Mo (V) complex with a maximal absorption at 695 nm. The phosphomolybdate method is a quantitative since the total antioxidant activity is expressed as AA equivalent [18]. The APE showed the highest total antioxidant activity (181.16 ± 35.727 mg equivalents of ascorbic acid) followed by APM (102.76 ± 19.57 mg equivalents), APC (89 ± 1.67 mg equivalents), APD (87.79 ± 8.00 mg equivalents) and APA (51.61 ± 19.72 mg equivalents) (Figure 4). APP did not show any activity and data are not shown.

### Determination of total phenolic content

**Figure 5:**
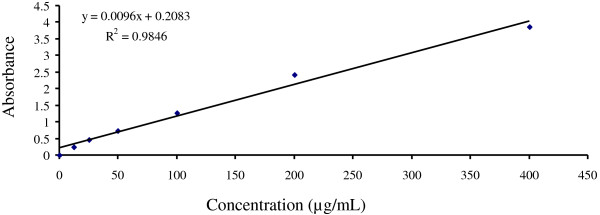
**Calibration curve of gallic acid.** Each point represents the mean of three experiments.

**Figure 6:**
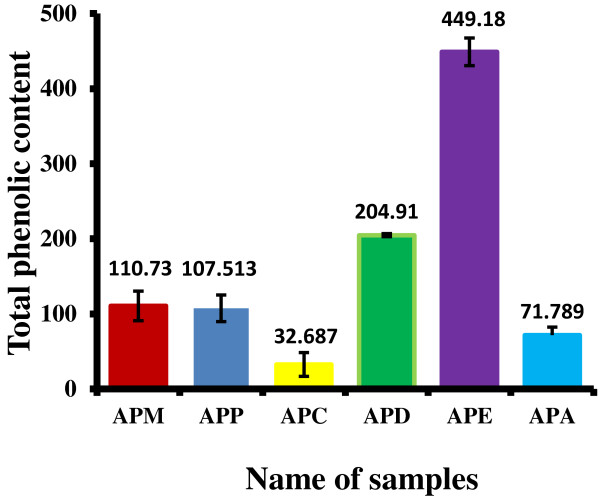
**Total phenolic content of APM and its derived fractions (APC, APD, APE and APA).** Values expressed are mean ± standard deviation (n = 3). The total phenolic contents are expressed as mg of gallic acid equivalent (GAE) per gm of extract. APM = Methanol extract, APC = Carbon tetrachloride fraction, APD = Dichloromethane fraction, APE = Ethyl acetate fraction, and APA = Aqueous fraction.

Total phenolic content was estimated by gallic acid (Figure 5) and expressed as milligrams of gallic acid equivalent (GAE). All the extracts contained a considerable amount of phenolic contents from 71.789 ± 10.46241 to 449.18 ± 18.41mg of GAE/g of extract (Figure 6). APE exhibited the highest total phenolic contents followed by APD, APM, APA, and APC.

### Phytochemical screening

The phytochemical screening of APM, APP, APC, APD, APE, and APD showed the presence of different types of secondary metabolites, namely saponins, tannins, glycosides and flavonoids (Table 2). These phytocompounds were present in almost all the extracts tested. However, APP showed the presence of only steroids and APC displayed the presence of only glycosides and flavonoids.

**Table 2:** **Phytochemical screening tests of leaves extracts of*Albizia procera***

**Samples**	**Saponins**	**Tanins**	**Glycosides**	**Steroids**	**Alkaloids**	**Flavonoids**
APM	**+**	**+**	**+**	-	–	**+**
APP	–	–	–	**+**	–	–
APC	–	–	**+**	–	–	**+**
APD	**+**	**+**	**+**	–	–	**+**
APE	**+**	**+**	**+**	–	–	**+**
APA	**+**	**+**	**+**	–	–	**+**

APM = Methanol extract, APP = Petroleum ether fraction, APC = Carbon tetrachloride fraction, APD = Dichloromethane fraction, APE = Ethyl acetate fraction and APA = Aqueous fraction.

Here, (+) = present and (–) = Absent.

## Discussion

Free radicals are known to play a definite role in a wide variety of pathological manifestations. Antioxidants fight against free radicals and protect us from various diseases. They exert their action either by scavenging the reactive oxygen species or protecting the antioxidant defense mechanisms [19].

In this study, the methanol extract and its derived fractions at various concentrations were tested for their antioxidant activity using DPPH radical scavenging assay, and reducing power capacity method. The results of both tests were positive. In addition, other tests such as phytochemical screening and total phenolic contents were also conducted.

The DPPH test is a widely used method to evaluate the free radical scavenging effect of plant extracts. This method is based on the reduction of methanolic DPPH solution in the presence of antioxidant resulting in the formation of non radical DPPH-H by the reaction.

The stable DPPH were reduced by all the extracts and, thus changing the color from purple to yellow to varying degree depending on the presence of antioxidant compounds. The degree of discoloration indicates the scavenging potential of the extract. In the present study, among all the extracts tested, the highest capacity to neutralize DPPH radicals was found for the APE and a moderate activity was found for other extracts.

Yutana *et al*. [10] used the ethanol to extract the stem bark of *Albizia procera*. They have not fractionated the ethanol extract with different solvent of different polarity. Their extract showed potent antioxidant activity in DPPH scavenging model when compared with ascorbic acid. They also described that this plant is used for tonic and longevity in Thailand. Here, we have used methanol for extraction of leaves instead of ethanol. Methanol has been known more effective to dissolve active compounds in cells. Hence, it was easier to penetrate the cellular membrane to extract the intracellular ingredients from plant materials. Tiwari *et al*. [20] stated that several active compounds will be obtained if methanol used as solvent in the extraction technique i.e. anthocyanins, saponins, tannins, flavones and polyphenols. It has also been reported that antioxidant activity of extracts is strongly dependent on the types of solvent used due to compounds with different polarity exhibiting differing rates of antioxidant potential [18]. So, the difference in the DPPH radical scavenging activity in different fractions implies that the extracting solvent used would affect the radical scavenging potency. Therefore, in addition to potent free radical scavenging activity of stem bark, leaves of *A. procera* may be a promising antioxidant which can protect against a wide range of free radical-induced diseases.

Previous reports suggested that the reducing properties have been shown to exert antioxidant action by donating of a hydrogen atom to break the free radical chain [21]. The antioxidants present in the APM and other fractions of *A*. *procera* leaves caused their reduction of Fe^3+^-ferricyanide complex to the ferrous form, and thus proved the reducing power. The ferric reducing power activity of APM and other fractions seem to be due to presence of polyphenols. The reducing capacity of plant extract may serve as a significant indicator of its potential antioxidant activity. Seddik *et al.* reported that the plant extract having reducing power can prevent liver injury by inhibiting the formation of lipid peroxides [22]. Like the DPPH radical scavenging activity, the reducing power of APM, APD, APC, APE and APA increased with increasing concentration.

Phenolic compounds are very important plant constituents because their hydroxyl groups confer scavenging ability [23]. Phenolics present in leaves have received considerable attention because of their potential antioxidant activities [24]. Plant materials rich in phenolics are increasingly being used in the food industry because they retard oxidative degradation of lipids and improve the quality and nutritional value of food [25]. Antioxidant activity of extracts is strongly dependent on the solvent due to the different antioxidant potentials of compounds with different polarity [26]. In this research, amongst the six extracts, the APE exhibited the highest total phenolic content and its greater radical scavenging and reducing capacity may be due to this higher content of phenolic compounds. Thus, the therapeutic properties of *A. procera* leaves may be possibly attributed to the phenolic compounds present.

The phytochemical tests indicated the presence of saponins, tannins, glycosides, and flavonoids in the extracts. Compounds of such classes are known to possess potent antioxidant activity [27]. However, the chemical constituents present in the extract, which are responsible for this activity still have not been reported and need to be investigated.

## Conclusion

The results of this study showed that leaves of *A. procera* contain phenolic compounds that contribute in the determination of total phenolic contents. The highest antioxidant activity and total phenolic contents were exhibited by the ethyl acetate fraction. This extract can be used to prevent diseases associated with free radical but proper animal toxicity studies are needed to validate possible clinical use of the extract.

## Abbreviations

AA: Ascorbic acid; APA: Aqueous fraction; APC: Carbon tetrachloride fraction; APD: Dichloromethane fraction; APM: Methanol extract; APP: Petroleum ether fraction; DPPH: 1, 1-diphenyl-2-picrylhydrazine; GAE: Galic acid equivalent.

## Competing interests

The authors declare that they have no competing interests.

## Authors’ contributions

MMK prepared the extracts and carried out all the experimental process. MSP designed the current project, supervised the work and wrote the manuscript. MEI worked closely with MMK in the laboratory to carry out the experiments. MRI, PK and MMR evaluated the data and edited the manuscript. MAAR did the phytochemical screening of the extracts. AHMKA participated in statistical analysis. All the authors read and approved the final manuscript.
